# Abnormal cerebellar connectivity and plasticity in isolated cervical dystonia

**DOI:** 10.1371/journal.pone.0211367

**Published:** 2019-01-25

**Authors:** Paolo Porcacchia, Paloma Álvarez de Toledo, Antonio Rodríguez-Baena, Juan Francisco Martín-Rodríguez, Francisco J. Palomar, Laura Vargas-González, Silvia Jesús, Giacomo Koch, Pablo Mir

**Affiliations:** 1 Unidad de Trastornos del Movimiento, Servicio de Neurología y Neurofisiología Clínica, Instituto de Biomedicina de Sevilla, Hospital Universitario Virgen del Rocío/CSIC/Universidad de Sevilla, Seville, Spain; 2 Unidad de Neurofisiología Clínica, Servicio de Neurología y Neurofisiología Clínica, Hospital Universitario Virgen del Rocío, Seville, Spain; 3 Clinica Neurologica, Dipartimento di Neuroscienze, Università di Roma Tor Vergata, Rome, Italy; 4 Centro de Investigación Biomédica en Red sobre Enfermedades Neurodegenerativas (CIBERNED), Seville, Spain; University of Toronto, CANADA

## Abstract

There is increasing evidence that supports the role of the cerebellum in the pathophysiology of dystonia. We used transcranial magnetic stimulation to test the hypothesis that patients with cervical dystonia may have a disrupted cerebellar cortical connectivity at rest, and that cerebellar plasticity is altered too. We enrolled 12 patients with isolated cervical dystonia and 13 controls. A paired-pulse transcranial magnetic stimulation protocol was applied over the right cerebellum and the left primary motor area. Changes in the amplitude of motor evoked potentials were analysed. Continuous and intermittent Theta Burst Stimulation over the cerebellum was also applied. The effects of these repetitive protocols on cortical excitability, on intra-cortical circuits and on cerebellar cortical inhibition were analysed. In healthy subjects, but not in dystonic patients, a conditioning stimulus over the cerebellum was able to inhibit the amplitude of the motor evoked potentials from primary motor cortex. In healthy subjects continuous and intermittent cerebellar Theta Burst Stimulation were able to decrease and increase respectively motor cortex excitability. Continuous Theta Burst Stimulation was able to abolish the cerebellar cortical inhibition observed in basal condition. These effects were not observed in patients with cervical dystonia. Cerebellar cortical connectivity and cerebellar plasticity is altered at rest in patients with cervical dystonia.

## Introduction

The involvement of the cerebellum in the pathophysiology of dystonia is a concept that has emerged over the last few years. Although the same definition of isolated dystonia excludes cerebellar signs on clinical examination, it is well known that the cerebellum is involved in some forms of secondary dystonia [1] and that patients with degenerative cerebellar disorders can present dystonia as part of the clinical phenotype [2].

Furthermore, animal models, imaging and neurophysiological studies recently provided some new evidence supporting the involvement of the cerebellum in some aspects of dystonia. For instance, studies in animal models showed that the injection of glutamatergic agonists such as kainic acid into the cerebellar vermis of mice, elicits dystonic postures of the trunk and limbs, while transgenic mice lacking Purkinje cells exhibit less dystonic movements [3]. Purkinje cells were clearly related to the severity of the dystonic episodes in tottering mice too [4] and in a knock-out mouse model of DYT1 dystonia, subtle structural changes were present in the cerebellum [5]. It is worth remembering the probable presence of a short-latency cerebellum modulation of the striatum [6] in mice. Under physiological conditions, this pathway permits a rapid communication between cerebellum and striatum, allowing a real-time modulation of muscle activity. But, under pathological conditions, it can cause movement disorders like dyskinesia and dystonia.

Imaging studies in humans showed how integrity of the cerebellar-thalamo-cortical fiber tract was reduced in both manifesting and clinically non-manifesting DYT1 and DYT6 dystonia mutation carriers and this finding was related to stronger motor activation responses [7]. In a study of network analysis, using positron emission tomography, the normal motor-related pattern topography (that included the cerebellum) was abnormal in DYT1 manifesting carriers [8]. High resolution magnetic resonance imaging in cervical dystonia revealed an increase in grey matter density of the cerebellar flocculus [9]. Voxel-based morphometry analysis showed, also in writer’s cramp, structural cerebellar abnormalities, in terms of grey matter decrease [10]. An alteration of cerebellar-thalamo-cortical pathway, in cervical dystonia was demonstrated using functional magnetic resonance and activation analysis [11].

Some studies have addressed this cerebellar involvement from a neurophysiological point of view. Eyeblink conditioning paradigm has been widely used to assess the function of the cerebellum and its associated circuitry. Abnormal responses in this paradigm were demonstrated in primary focal dystonia [12]. This result was confirmed in another experiment with patients with cervical dystonia and, interestingly, cerebellar continuous theta burst stimulation (cTBS) was able to modulate eyeblink conditioning in these patients towards a normalization [13]. In contrast, circuits involved in eyeblink conditioning were found to be normal in DYT1 and DYT6 dystonia, even if the blink reflex recovery cycle, a marker of brainstem excitability, was reduced in the DYT1 subtype [14]. In addition, eyeblink conditioning was normal in patients with secondary dystonia [15]. In patients with focal upper limb dystonia, transcranial magnetic stimulation (TMS) applied over cerebellum failed to modulate the excitability of the primary motor cortex as in controls [16]. To summarize these pieces of evidence, we can affirm that the cerebellum is probably involved in the pathophysiology of dystonia as a part of the altered, underlying motor circuit. However, some contrasting findings may reflect an early stage of the investigation in this field, as well as the need to differentiate the different subtypes of dystonia.

At the end of this short review of the literature, we will focus our attention on cerebellar plasticity. In patients with writer’s cramp, the effect of intermittent and continuous cerebellar Theta Burst Stimulation (iTBS and cTBS) on paired associative stimulation (PAS) and on other cortical excitability parameters were studied [17]. It was found that, unlike the control group, PAS-induced plasticity was longer-lasting in patients and did not change after cerebellar cTBS or iTBS. Anodal cerebellar transcranial direct current stimulation, in writer’s cramp patients, failed to modulate electrophysiological and clinical parameters and patients exhibited a large variability in the direction and size of their plasticity responses [18]. In a recent study, we demonstrated, in patients with cervical dystonia, that 10 days of cerebellar cTBS were able to produce a clinical improvement and to restore a normal pattern of topographic specific induced plasticity [19].

So, at present, we know that cerebellar plasticity in dystonia is probably altered but, in some way, it can be eventually modulated. But what is “abnormal”? Is it simply a weaker effect compared to normal subjects or is it a different effect? In other terms, does an inhibitory TMS repetitive protocol have a smaller effect on dystonia or does it have an excitatory effect (and vice versa)? Answering these questions has a significant clinical implication when drawing an intervention to modulate an altered cortical excitability. In the present study, to better understand the pattern of cerebellar cortical plasticity in patients with cervical dystonia, we decided to study the effects of both cerebellar cTBS and iTBS protocols on the excitability of primary motor cortex (M1), as well as to study cerebellar cortical connectivity in basal conditions.

## Methods and materials

### Subjects

Twelve right-handed patients (6 men, 6 women, mean age 47 ± 8 years, disease duration 8 ± 5 years) affected by isolated cervical dystonia (Table 1) were recruited from the Movement Disorders Outpatient Clinic at the “Hospital Universitario Virgen del Rocío” in Seville, Spain. Diagnosis of cervical dystonia was made by expert neurologists, based on medical history and clinical examination. The assessment included a complete Toronto Western Spasmodic Torticollis Rating Scale (TWSTRS) and the Burke-Fahn-Marsden Dystonia Rating Scale (BFMDRS). The TMS experiments were performed at least 3 months after the last botulinum toxin injection. All other oral drugs were stopped 48 h before the TMS experiments.

**Table 1:** Clinical characteristics of patients with cervical dystonia.

N°	Sex	Age (years)	Disease duration (years)	TWSTRS	BFMDRS	Dominant hand/ Head deviation	Treatment (mg/day)
1	F	56	3	13	1.5	R/R	BT
2	F	51	6	26.75	6	R/L	BT
3	M	60	8	13.75	2.5	R/L	/
4	F	48	8	25	39.5	R/L	BT
5	M	44	8	25.50	4	R/R	BT
6	F	45	8	48.25	7.5	R/L	BT
7	M	36	11	40.75	17.5	R/R	BT
8	F	54	4	42.25	13	R/L	Clonazepam (0.5)
9	F	48	8	22.50	7.5	R/L	BT
10	M	38	11	64	40	R/R	Clonazepam (2)
11	M	49	22	22.75	4	R/L	BT
12	M	33	2	30.25	5	R/L	BT

M: male, F: female, R: right, L: left, BT: botulinum toxin, TWSTRS: Toronto Western Spasmodic Torticollis Rating Scale, BFMDRS: Burke-Fahn-Marsden Dystonia Rating Scale.

Thirteen age-matched (6 men and 7 women, 45 ± 9 years), healthy, (11 right-handed and 2 left handed) volunteers served as control subjects. They were recruited from the hospital and research staff.

The study was approved by the local ethics committee (Ethics and Research Committee of the University Hospital “Virgen del Rocío”, Seville-"Subcomisión de la Investigación Sanitaria del Hospital Virgen del Rocío: acta 09/2010”) and all the subjects gave written informed consent. All experiments were performed in accordance with relevant guidelines and regulations.

### Experimental design

The study was completed in two different sessions, performed at least one week apart, for both patients and controls. In each session, a hot spot for contralateral first dorsal interosseus (FDI) was found and marked on the scalp. We measured resting motor threshold (RMT), active motor threshold (AMT) [20, 21], and stimulation intensity to evoke a motor evoked potential (MEP) amplitude of approximately 1 mV.

In one session, we studied the effects of cerebellar iTBS of the lateral cerebellum on MEPs, short intracortical inhibition/facilitation (SICI-ICF) and cerebellar cortical inhibition (CBI) circuits. In the other session, the effects of cerebellar cTBS on the same variables were studied. A random order was used for choosing whether to apply cerebellar iTBS first or cTBS.

In both sessions, baseline measures of MEP amplitude (20 stimuli), SICI-ICF and CBI were performed, then cerebellar TBS (continuous in one session and intermittent in the other one) was delivered. Immediately after the repetitive stimulation (t0) and 20 minutes after (t20), we repeated the measures of MEP, SICI/ICF and CBI. MEP amplitude protocol was repeated a third time, 40 minutes after repetitive stimulation (t40). MEP amplitudes, measured in the right FDI muscle, were analysed (we used left FDI for the two left handed subjects). EMG signals were amplified (1000) and band-pass filtered (bandwidth 30 Hz-1 kHz) with a Digitimer D360 amplifier (Digitimer, UK), acquired at a sampling rate of 5 kHz through a CED 1401 laboratory interface (Cambridge Electronic Design, Cambridge, UK) and then recorded by a computer using SIGNAL software for an off-line analysis.

Subjects were seated comfortably, looking forward, with the head in a neutral position during the experiment. We asked the subjects to immediately alert if they note a cervical contraction or, in general, if they were not able to maintain the neutral position of the head. If so, or if the researcher perceived a cervical or head movement, the TMS series was stopped and started again.

### MEPs protocol

TMS was performed with a MagStim 200 (Magstim Co., Whitland, Dyfed, UK) connected to a standard figure-of-eight flat coil (70 mm diameter) placed over the primary motor cortex. Over the motor ‘hot spot’, 20 test stimuli (TS) were delivered with a 5 seconds inter-trial interval and resultant MEPs were recorded. Stimulation intensity was set to obtain a MEP of 1 mV, with the muscle relaxed. The same intensity was used, after repetitive stimulation, at time 0, after 20 minutes and 40 minutes (t0, t20 and t40).

### SICI/ICF protocol

TMS was performed with two MagStim 200 connected with a BiStim module (Magstim Co., Whitland, Dyfed, UK) to a standard figure-of-eight flat coil (diameter 70 mm) placed over primary motor cortex. The coil delivered two stimuli: a cortical conditioning stimulus (CS) and a test stimulus (TS). Intensity of CS was set at the 80% of AMT. The CS preceded the TS by seven different inter stimulus interval (ISI): 1, 2, 3, 5, 7, 10, 15 ms. There were eight conditions corresponding to the seven different ISI and the TS alone. Ten responses were collected for each different ISI condition and 10 responses were collected with TS alone (80 trials in total). All conditions were performed in a random order. TS intensity was set to obtain a MEP of 1 mV, with the muscles relaxed. The intensity of TS could be adjusted after repetitive protocol to obtain 1 mv MEP amplitude in each series.

### CBI protocol

TMS was performed with two MagStim 200 connected to two standard figure-of-eight flat coils (70 mm diameter). One coil stimulated primary motor cortex. The second coil was used to deliver the CS and it was placed over the contralateral cerebellar hemisphere, 3 cm lateral, and 1 cm caudally from the inion [22, 23].

We set the intensity for the motor cortical TS at the intensity that produced MEP of 0.5–1 mV, with the muscle relaxed. The CS preceded the TS by different ISI ranging from 3 to 15 ms (3, 5, 7, 9, 15 ms). There were six conditions, corresponding to the five different ISI and the TS alone. Ten responses were collected for each different ISI conditions and 10 responses were collected with TS alone (60 trials in total). The order of the conditions was random. We studied right cerebellar-left cerebral hemisphere connection. In the two left-handed subjects, we studied left cerebellar- right cerebral hemisphere connection. This decision took into account the dominant hemisphere in all patients and control subjects. CS intensities were set at 90% of RMT obtained in the ipsilateral motor cortex. The intensity of TS could be adjusted after repetitive protocol to obtain 0.5–1 mv MEP amplitude in each series.

### Repetitive stimulation

Continuous and intermittent Theta Burst Stimulation (cTBS and iTBS) were performed with a MagStim Super Rapid magnetic stimulator (Magstim Co., Whitland, Dyfed, UK) connected to a standard figure-of-eight flat coil (70 mm diameter) placed over the right cerebellar hemisphere, as in previous studies [22, 24]. The intensity of the repetitive stimulation was set at 80% of the AMT [21].

### Data analysis

The Shapiro-Wilk test was used to check the normal distribution of the data. Parametric or non-parametric tests were used for data with or without normal distribution respectively. Magnetic stimulation intensities, clinical and demographic data were analysed using Wilcoxon and Mann-Whitney U tests, depending on data type.

To study SICI/ICF and CBI in the basal conditions, for each subject, the mean values obtained before iTBS and cTBS were calculated, and paired t-test was used between patients and controls.

To study the effects of cerebellar cTBS and iTBS on MEP, SICI/ICF and CBI, we utilized the following procedure. First, in each group (controls or patients), raw data were analysed using ANOVA with “TIME” (before i-cTBS, t0, t20, t40) as the within-subject factor. In this manner we explored the effects of the repetitive protocol (cerebellar cTBS or iTBS) on cortical excitability (MEP), on intracortical circuits (SICI/ICF) and on cerebellar cortical connectivity (CBI), in each group. Then to study the differences between groups repeated measures ANOVA was used. In MEPs study, data were analysed using repeated measures ANOVA, with “TIME” as the within-subject factors and “GROUP” (patients vs. controls) as the between-subject factor. In SICI/ICF and CBI experiments, conditioned MEP amplitudes were normalized to a non-conditioned one (Test alone). Normalized data was analysed using repeated measures ANOVA, with “TIME” and “ISI” (1, 2, 3, 5, 7, 10, and 15 ms for SICI/ICF and 3, 5, 7, 9, and 15 ms for CBI) as the within-subject factors and “GROUP” (patients vs. controls) as the between-subject factor. A significant interaction in the ANOVA was followed by post-hoc paired t-test analysis with Bonferroni correction. The Greenhouse-Geisser correction was used for non-spherical data and Mauchly’s test examined for sphericity. Pearson’s and Spearman’s correlations, between clinical scale scores and neurophysiological data were performed to explore the clinical to functional relationship. The neurophysiological data, used to seek for clinical to functional relationship, were CBI and SICI baselines values (at 5 ms ISI) and mean value of normalized MEP ((t0 amplitude + t20 amplitude + t40 amplitude)/3) both after cTBS and iTBS.

A P value of <0.05 was considered as statistically significant in all analyses. All statistical analyses were carried out using IBM SPSS Statistics 20 software.

## Results

### Demographic data and magnetic stimulation intensities

No significant differences were found in demographic data between patients and control subjects. RMT, AMT and intensity for repetitive stimulation were not different among groups. RMT was 37.8% ± 5.6 (of maximal stimulator output) in the control group and 41.3% ± 8.1 in patients (P = 0.17). AMT was 30.3% ± 3.7 in the control group and 33.2 ± 5.2 in patients (P = 0.13). Intensity for repetitive stimulation was 33.1% ± 5.1 in the control group and 35.0% ± 4.9 in patients (P = 0.32).

### SICI-ICF

**Fig 1:**
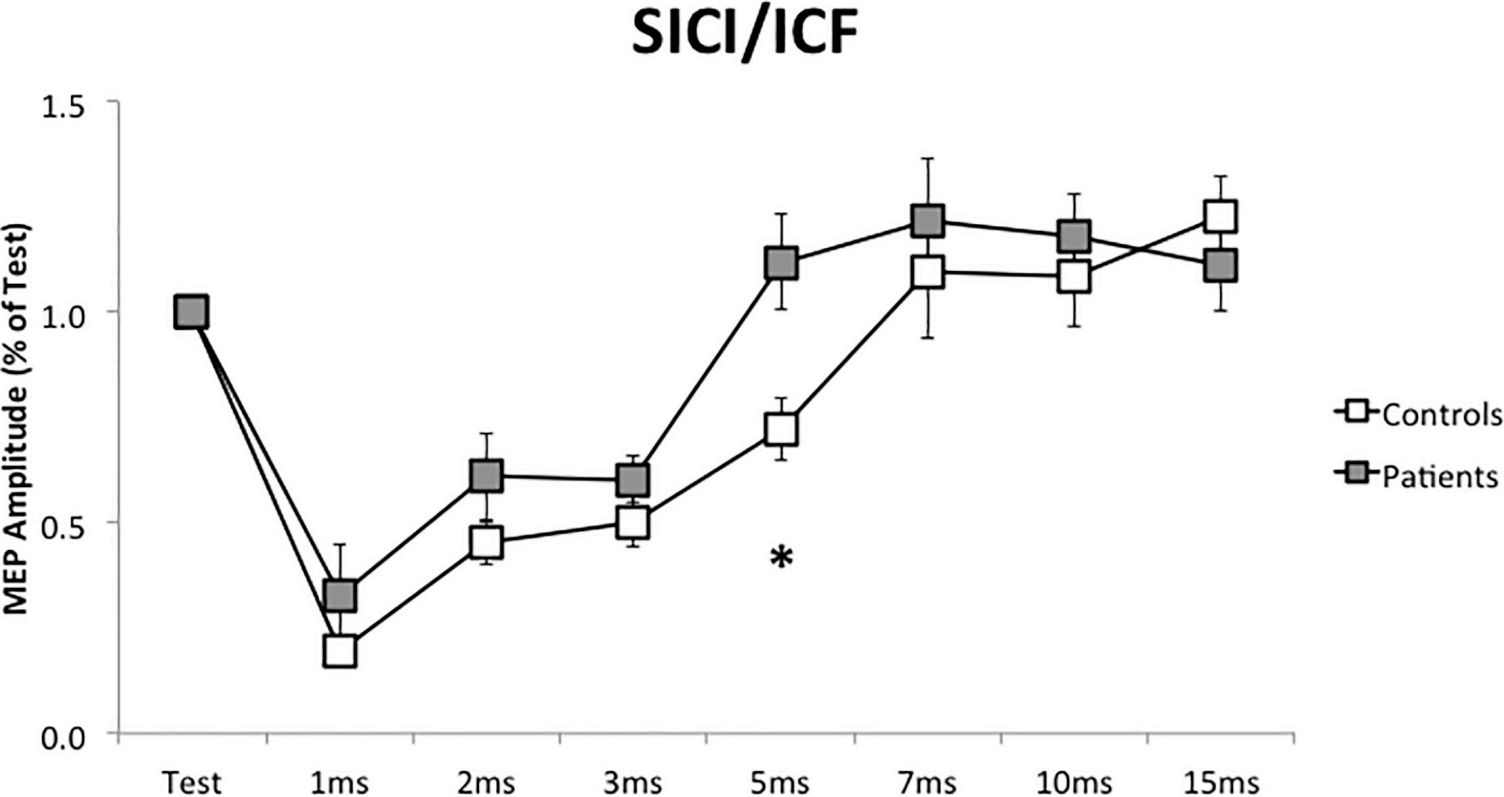
Short intracortical inhibition/intracortical facilitation at baseline, before the repetitive stimulation (SICI/ICF). SICI/ICF was performed before cerebellar iTBS and cTBS. In each group, mean values of the two measurements were calculated. Horizontal axis: Test stimulus and the different inter-stimuli intervals. *: P < 0.05. Error bars represent the standard error.

At baseline, before iTBS or cTBS protocols, controls and patients differ in MEP amplitude at 5 ms ISI (P = 0.014, Fig 1). In control subjects, the conditioning stimulus was able to inhibit MEP amplitude (0.74 of Test) but in dystonic patients, there was no inhibition (MEP amplitude 1.13 of Test). In each subject, baseline measurements (before iTBS or cTBS protocols) were not different.

To explore the clinical to functional relationship, we evaluate a possible relation between baseline amplitude at 5 ms interval and clinical scale, in patients. We did not find a significant relation.

### CBI

**Fig 2:**
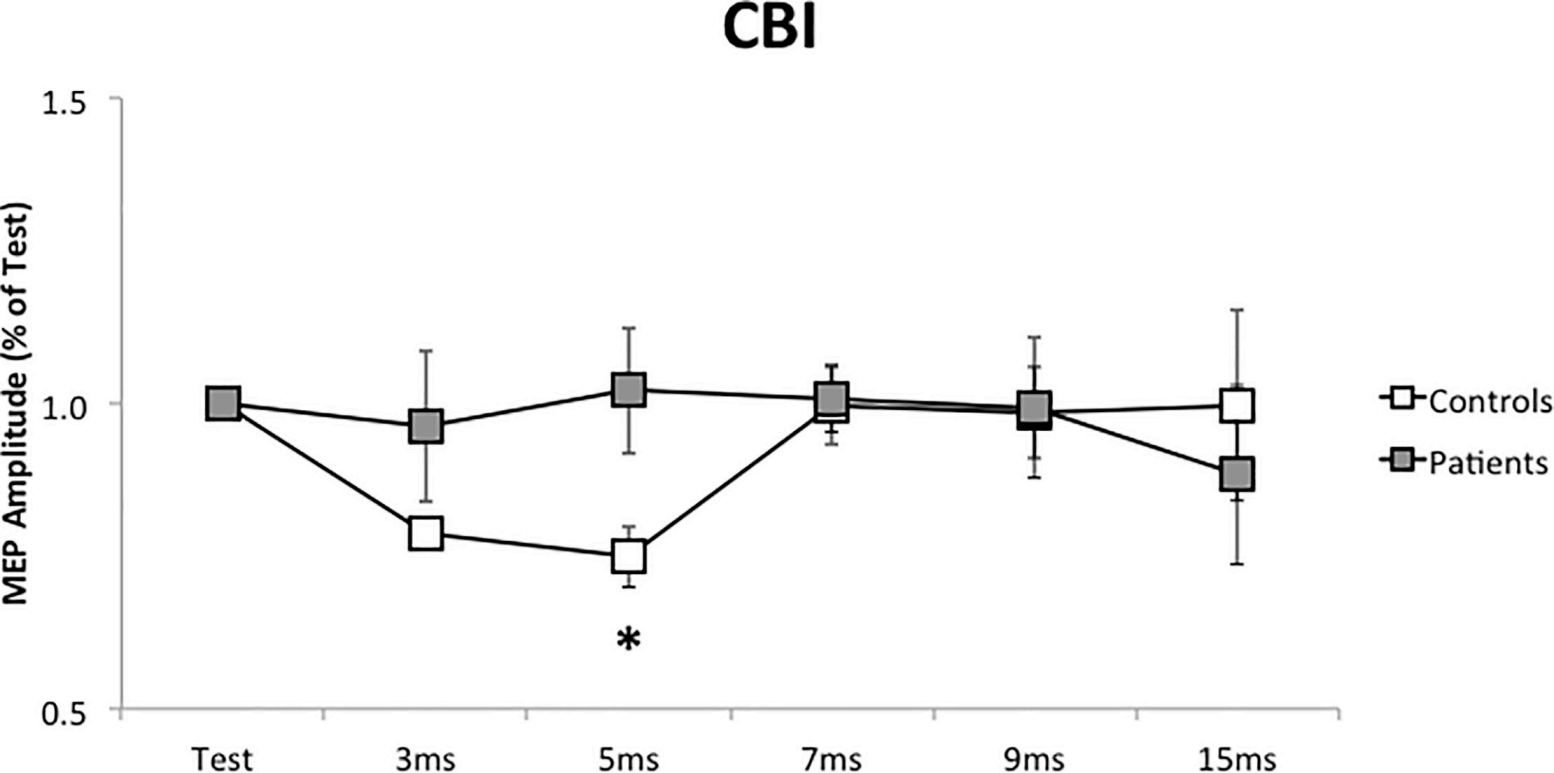
Cerebellar cortical inhibition (CBI) at baseline, before the repetitive stimulation. CBI was performed before cerebellar iTBS and cTBS. In each group, mean values of the two measurements were calculated. Horizontal axis: Test stimulus and the different inter-stimuli intervals. *: P < 0.05. Error bars represent the standard error.

Patients did not show cerebellar inhibition at baseline, as observed in control subjects. At 5 ms ISI, MEP amplitude in controls was 0.75 of Test, while in patients was 1.01 (P = 0.009, Fig 2). In each subject, baseline measurements (before iTBS or cTBS protocols) were not different.

When we evaluate a possible relation between baseline amplitude at 5 ms interval and clinical scale, in patients, we did not find a significant relation. However, there was a trend in which more inhibition (lower CBI values) was associated with lower clinical scale scores. In other terms, the permanence of cerebellar cortical connectivity was associated with a better clinical condition.

### Cerebellar iTBS

#### MEP protocol

**Fig 3:**
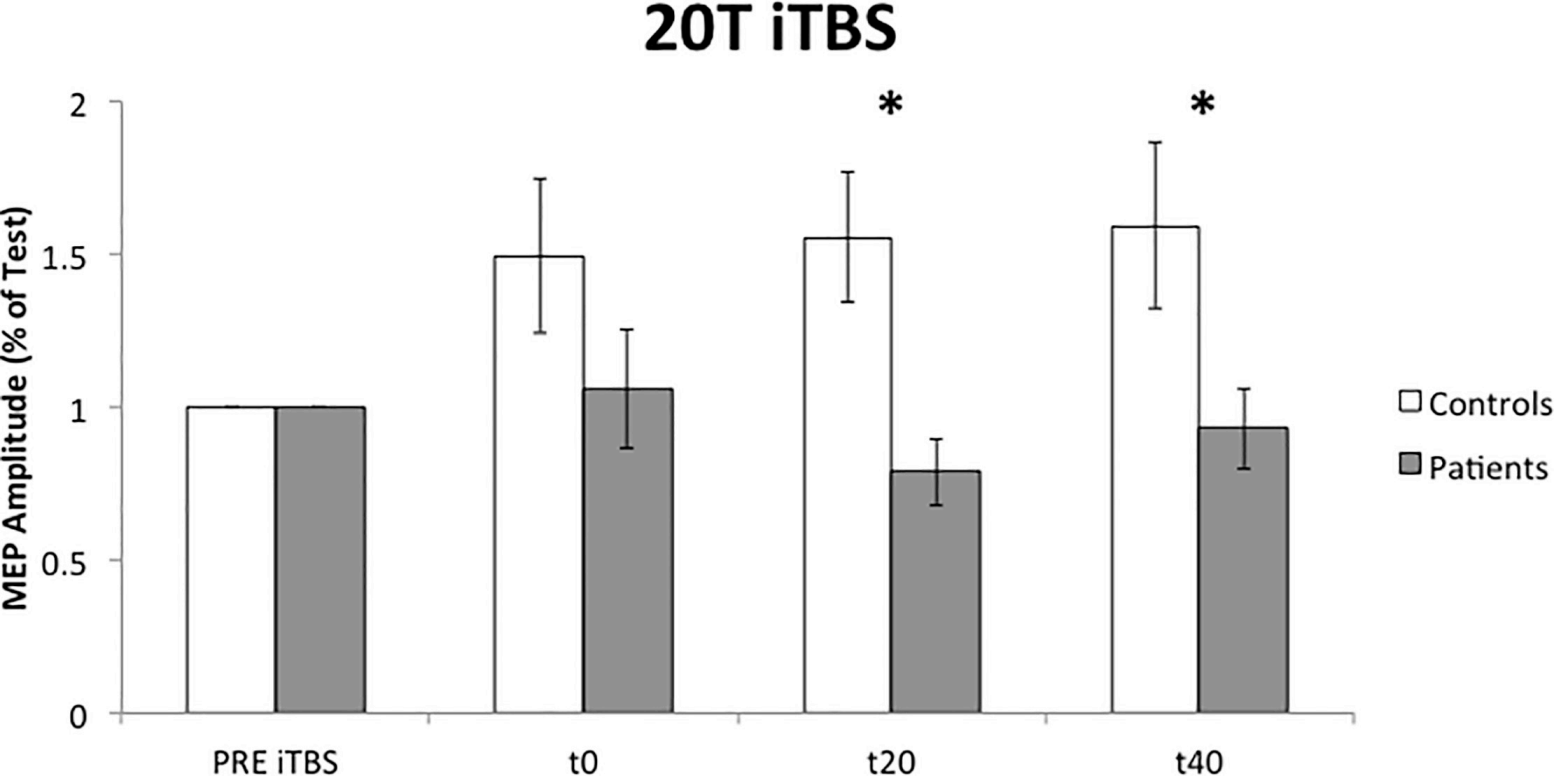
Effects of cerebellar iTBS on cortical excitability (MEP protocol). 20T: 20 Test. PRE: before iTBS; t0: immediately after iTBS; t20: 20 minutes after iTBS; t40: 40 minutes after iTBS. *: P < 0.05. Error bars represent the standard error.

iTBS had a significant effect on MEP amplitude in control subjects (P = 0.024, F = 6.706) but not in dystonic group. Repeated measures ANOVA revealed a significant interaction of “TIME*GROUP” (P = 0.024, F = 3.852). In fact, control subjects showed a facilitation of MEP amplitude after cerebellar iTBS (Fig 3) but patients did not. Paired t-test analysis showed a significant difference at t20 (P = 0.005) and t40 (P = 0.043).

No significant relation was found between clinical scales, in patients, and MEP amplitude variation after cerebellar iTBS.

#### SICI/ICF protocol

We observed no significant effects of cerebellar iTBS on intra-cortical circuits, assessed by SICI/ICF protocol (S1 Fig). In patients, we observed a trend to a reduction of intra-cortical facilitation (7, 10 and 15 ms) at t0.

#### CBI protocol

We observed no significant effects of cerebellar iTBS on cerebellar cortical inhibition (CBI), in either group (S2 Fig).

### Cerebellar cTBS

#### MEP protocol

**Fig 4:**
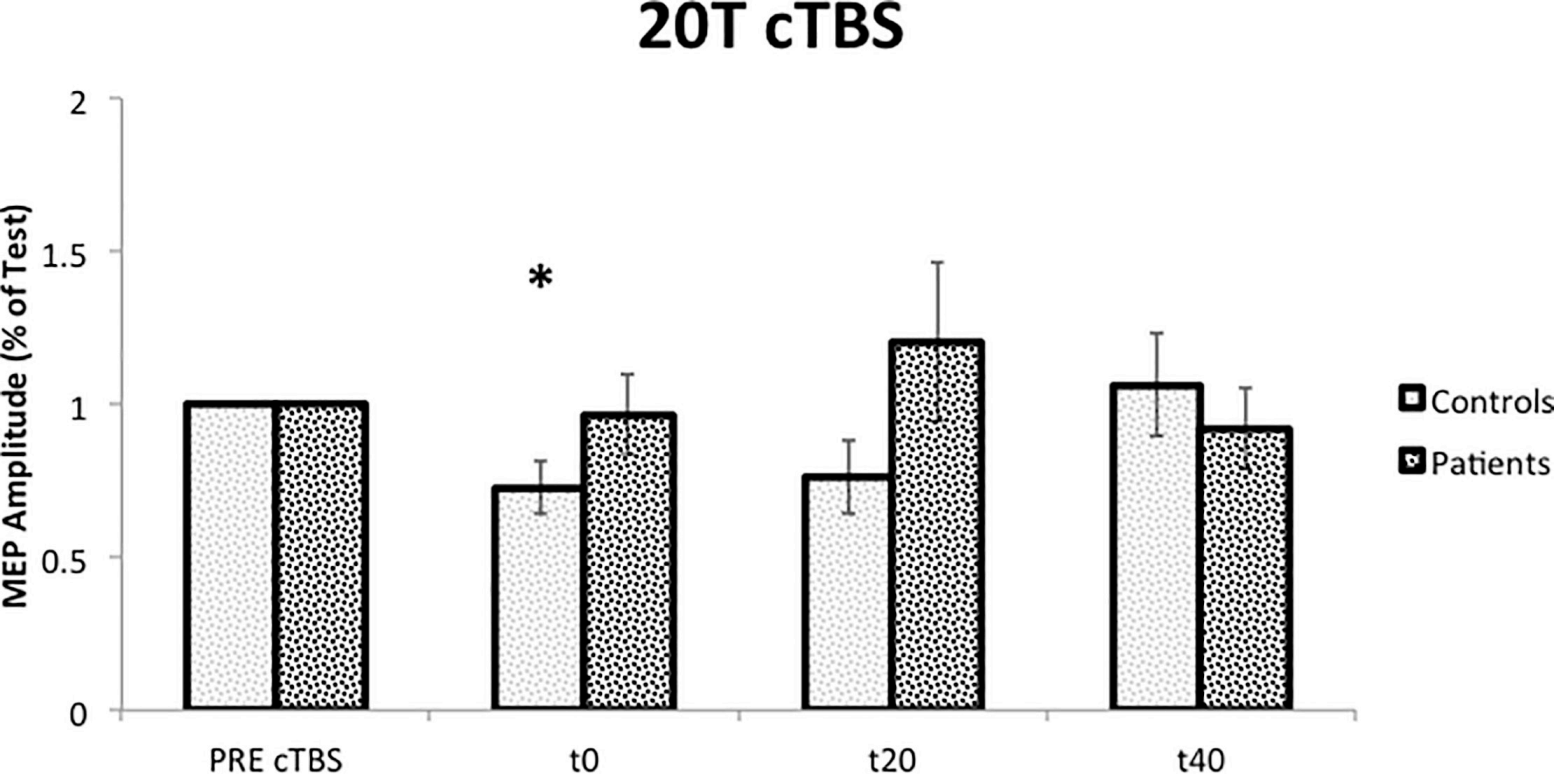
Effects of cerebellar cTBS on cortical excitability (MEP protocol). 20T: 20 Test. PRE: before cTBS; t0: immediately after cTBS; t20: 20 minutes after cTBS; t40: 40 minutes after cTBS. *: P < 0.05 (difference, in control group, compared to the value obtained before the cTBS). Error bars represent the standard error.

cTBS had a significant effect on MEP amplitude in control subjects (P = 0.029, F = 4.551), that reflected a MEP inhibition after cTBS at t0 (P = 0.046). This inhibition was not present in dystonic patients (Fig 4).

No significant relation was found between clinical scales, in patients, and MEP amplitude variation after cerebellar cTBS.

#### SICI/ICF protocol

We observed no significant effects of cerebellar cTBS on intra-cortical circuits, assessed by SICI/ICF protocol (S3 Fig).

#### CBI protocol

**Fig 5:**
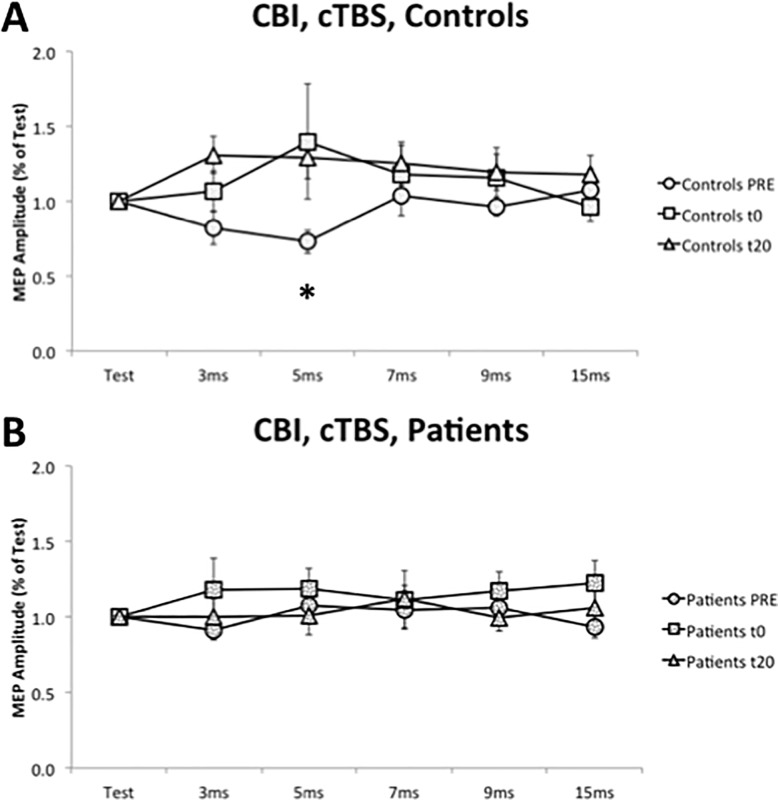
Effects of cerebellar cTBS on cerebellar cortical inhibition (CBI) in controls (A) and patients (B). PRE: before cTBS; t0: immediately after cTBS; t20: 20 minutes after cTBS. Horizontal axis: Test stimulus and the different inter-stimuli intervals. *: P < 0.05. Error bars represent the standard error.

Repeated measures ANOVA showed a significant main effect of “TIME” (P = 0.047, F = 3.157). Cerebellar inhibition, observed in the control group at baseline, was lost after cTBS. Paired analysis, between patients and controls presented a significant difference at baseline, at 5 ms ISI (P = 0.018), but not at t0 (P = 0.632) or at t20 (P = 0.168) (Fig 5).

## Discussion

The present study focuses the attention on the cerebellum and on its role in cervical dystonia. The study provides data about the function of the cerebellar-thalamo-cortical pathway, from at least two different perspectives. Firstly, the study of CBI, with a conditioning stimulus over cerebellum and the test stimulus over contralateral primary motor cortex, tells us about the basal functionality of this pathway, in terms of the current amount of the inhibition/facilitation of one structure (cerebellum) over the other (contralateral primary motor cortex). Secondly, the study of the effects of the cerebellar repetitive stimulation explores the potentiality of this pathway to be modulated, and the size and direction that this modulation can acquire, following two opposite protocols (cerebellar iTBS and cTBS). To study the plasticity of a pathway that include numerous structures and circuitries (cerebellum, thalamus, primary motor area), we provide data about three possible “targets” of the cerebellar iTBS or cTBS, which are primary motor cortex excitability (MEP amplitude), intracortical circuitry (SICI/ICF) and the cerebellar-thalamo-pathway itself (CBI).

The paradigm of cerebellar cortical inhibition has been well known for two decades [23]. Even if it was initially described with a double cone coil for cerebellar stimulation, we use, as in previous studies [22, 25], an experimental setting with two figure-of-eight flat coils. In the present study, we provide the evidence that patients with cervical dystonia had an abnormal CBI (Fig 2): in basal condition, a cerebellar conditioning stimulus is not able to reduce MEP amplitude in patients as in control subjects (5 ms). Brighina et al. [16], using TMS, already proved it in a group of writer’s cramp and musician dystonic patients and our study extends this finding to patients with cervical dystonia.

Both iTBS and cTBS protocols failed to facilitate or inhibit MEP amplitudes in dystonic patients as in control subjects. Our data suggests that dystonic patients had an abnormal cerebellar plasticity both in terms of the reduced effect of TBS and in terms of loss of specific effects (inhibition or increase depending on repetitive stimulation protocol). These results could have clinical implications. If we consider the ability to modify MEP amplitude as in controls, iTBS seems to have less power compared to cTBS and, probably, cerebellar iTBS is not the best candidate when choosing a TMS protocol for therapeutic purposes. Clinical significance of a two weeks cerebellar cTBS, in patients with cervical dystonia, was already demonstrated in previous research (Koch 2014). cTBS was able to produce a clinical change (assessed by TWSTRS) and to restore a somatotopic specificity in a paired associative stimulation protocol.

In the present study, cerebellar TBS was not able to modify intra-cortical circuits, assessed by SICI/ICF. In previous research performed on healthy subjects [24], cerebellar iTBS was able to reduce intra-cortical facilitation (ICF), while cerebellar cTBS was able to reduce short intra-cortical inhibition (SICI). Age differences between the two samples could explain this difference. In Koch’s research (2008), the age range was 20 to 37 years old, while in the present study, the mean age is over 40 years old, with a range that exceeds 60 years old. In fact, in the older population, Di Lorenzo et al. did not find the modulation of SICI following cTBS either. [26] The analysis of intra-cortical circuits is still more complex. Even if it was not an objective of the present study, one problem to address is whether the intra-cortical inhibition is altered or not in basal condition, in dystonic patients. In writer’s cramp patients, short intra-cortical inhibition was reduced [27, 28]. Conversely, Amadio et al. [29], in patients with cervical dystonia with sensory tricks, found that intra-cortical inhibition was only slightly and not significantly reduced, at rest, compared to control subjects. The last authors analysed short intra-cortical inhibition with two inter-stimuli intervals (1 and 3 ms). In our study, we obtained similar results at 1 and 3 ms (Fig 1) but we found a significant difference at 5 ms, supporting a lack of inhibition in the patient’s group. One important aspect of the present study is its internal reliability. All measurements were collected in basal conditions, before repetitive stimulation (RMT, AMT, MEP, SICI, CBI) and were repeated twice both in controls and patients, once for each session (iTBS or cTBS). In this context, the difference observed at 5 ms extends SICI alteration to patients with cervical dystonia and suggests similar neurophysiological mechanism in different types of dystonia (writer’s cramp and cervical dystonia). The next question is why do we obtain a difference at 5 ms and not at 1, 2 or 3 ms, in which there is a stronger inhibition. One possibility is that SICI alteration, even if present in different forms of dystonia could be more evident in some of these, such as focal hand dystonia, and less evident in others, such as cervical dystonia. Another possibility is that SICI alteration could have a somatotopic specificity. In this case, it should be easier to find a SICI alteration in writer’s cramp if we study a hand muscle (as is the case in the majority of the TMS studies). So, in patients with cervical dystonia, SICI alteration could be more evident in the somatotopic area that corresponds to cervical muscles and could be less evident when we placed the coil over the somatotopic area that corresponds to the first dorsal interosseus. However, SICI alteration was described bilaterally in writer’s cramp patients [27]. This point could be the object of future studies.

Finally, we examine the effects of repetitive stimulation on cerebellar cortical inhibition. In previous research on healthy subjects, Popa et al. demonstrated a significant suppression of CBI after cerebellar cTBS but not after cerebellar iTBS [30]. Our research replicates these results in control subjects, where the significant inhibition (at 5 ms ISI) was lost after cTBS. In patients, there is a lack of cerebellar inhibition at baseline, and there is no significant change after repetitive stimulation. This indicates that one session of iTBS or cTBS is not able to restore the cerebellar cortical connectivity in patients with cervical dystonia. In CBI protocol, we studied five different inter-stimuli intervals. We did not observe the inhibition at 5 ms, nor at a different inter-stimulus interval. So, we can affirm that neither physiological inhibition nor an aberrant inhibition exists in patients with cervical dystonia, both at baseline and after repetitive stimulation. Immediately after the iTBS, we observed in patients a non-significant trend towards a paradoxical facilitation (3 and 9 ms). For this reason, iTBS appears not to be suitable to restore a correct cerebellar cortical connectivity.

In conclusion, this paper provides evidence that patients with cervical dystonia had an abnormal cerebellar cortical connectivity at rest and that the plasticity of this pathway is also altered. Altered plasticity is evident both in terms of a reduced effect of cerebellar TBS and in terms of loss of specific excitability changes, when different plasticity protocols are applied. Furthermore, one session of cerebellar TBS was not able to restore a normal cerebellar cortical connectivity. From the point of view of the future clinical implications, cerebellar iTBS was more unpredictable, because its neurophysiological effects showed a major divergence compared to those obtained in controls. For these reasons, cerebellar iTBS is probably less appropriate in selecting a cerebellar stimulation protocol for therapeutic purposes.

## Supporting information

S1 Fig Effects of cerebellar iTBS on intra-cortical circuits (SICI/ICF) in controls (A) and patients (B). PRE: before iTBS; t0: immediately after iTBS; t20: 20 minutes after iTBS. Horizontal axis: Test stimulus and the different inter-stimuli intervals. Error bars represent the standard error.
(TIFF)

S2 Fig Effects of cerebellar iTBS on cerebellar cortical inhibition (CBI) in controls (A) and patients (B). PRE: before iTBS; t0: immediately after iTBS; t20: 20 minutes after iTBS. Horizontal axis: Test stimulus and the different inter-stimuli intervals. Error bars represent the standard error.
(TIFF)

S3 Fig Effects of cerebellar cTBS on intra-cortical circuits (SICI/ICF) in controls (A) and patients (B). PRE: before cTBS; t0: immediately after cTBS; t20: 20 minutes after cTBS. Horizontal axis: Test stimulus and the different inter-stimuli intervals. Error bars represent the standard error.
(TIFF)
