# Supplementary figures and images for: Abnormal cerebellar connectivity and plasticity in isolated cervical dystonia
Source: PLoS One. 2019 Jan 25;14(1):e0211367. doi: 10.1371/journal.pone.0211367 (PMC6347195; doi:10.1371/journal.pone.0211367)

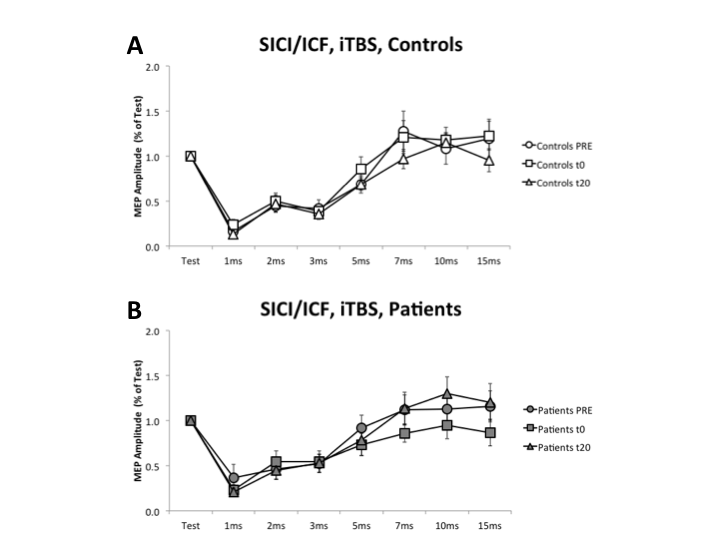

Supplement: S1 Fig — Effects of cerebellar iTBS on intra-cortical circuits (SICI/ICF) in controls (A) and patients (B). PRE: before iTBS; t0: immediately after iTBS; t20: 20 minutes after iTBS. Horizontal axis: Test stimulus and the different inter-stimuli intervals. Error bars represent the standard error. (TIFF) [file pone.0211367.s001.tiff]

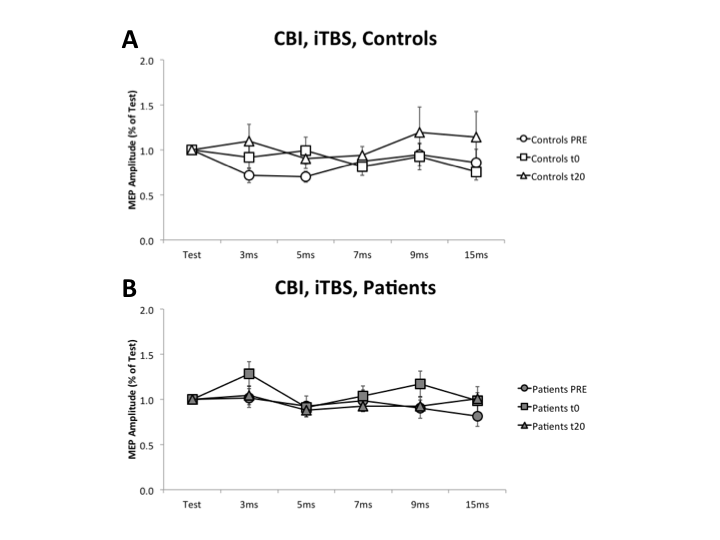

Supplement: S2 Fig — Effects of cerebellar iTBS on cerebellar cortical inhibition (CBI) in controls (A) and patients (B). PRE: before iTBS; t0: immediately after iTBS; t20: 20 minutes after iTBS. Horizontal axis: Test stimulus and the different inter-stimuli intervals. Error bars represent the standard error. (TIFF) [file pone.0211367.s002.tiff]

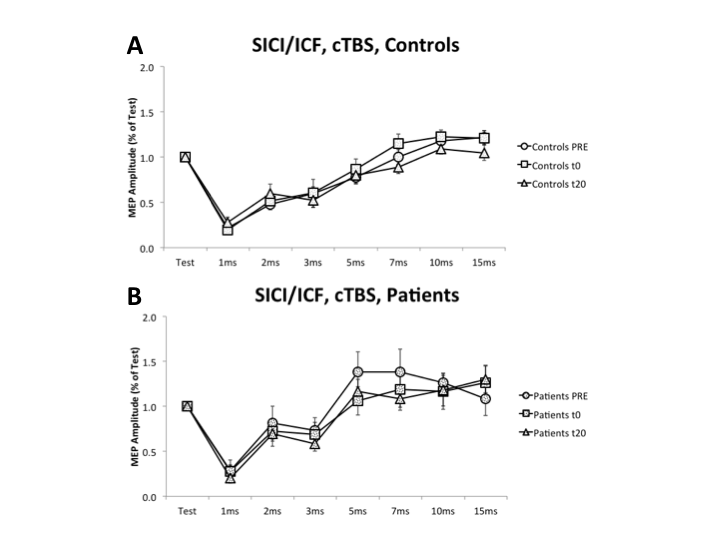

Supplement: S3 Fig — Effects of cerebellar cTBS on intra-cortical circuits (SICI/ICF) in controls (A) and patients (B). PRE: before cTBS; t0: immediately after cTBS; t20: 20 minutes after cTBS. Horizontal axis: Test stimulus and the different inter-stimuli intervals. Error bars represent the standard error. (TIFF) [file pone.0211367.s003.tiff]
